# Early weaning in pigeons (*Columba Livia domestica*): effects on squabs performance and reproductive performance of parents

**DOI:** 10.1186/s12917-025-04696-x

**Published:** 2025-04-14

**Authors:** Omar A. Mohamed, Ibrahim M. Khattab, Mohammad A. Elsagheer

**Affiliations:** 1 Department of Poultry Production, Faculty of Desert and Environmental Agriculture, Matrouh University, Matrouh, Egypt; 2 Department of Animal and Fish Production, Faculty of Desert and Environmental Agriculture, Matrouh University, Matrouh, Egypt; 3 Animal Production Department, Faculty of Agriculture, Azhar University, Assiut, Egypt

**Keywords:** Early weaning, Pigeons, Squab, Hand feeding, Growth performance, Reproductive performance

## Abstract

**Background:**

Artificial hatching and early artificial feeding of squabs can potentially reduce pigeon rearing costs, shorten breeding cycles, and enhance overall productivity. Two experiments were conducted to evaluate the effect of early weaning on growth performance, feed consumption, parents’ reproductive performance, and egg traits.

**Methods:**

In Experiment 1, A total of 300 pairs of adult White Mirthys pigeons were randomly divided into three groups: W28, W7, and W0. These groups represent pigeons separated from their squabs at different ages: 28 days, 7 days, and 0 days (at hatch), respectively. Each group consisted of ten replicates, with each replicate comprising ten pairs of pigeons. In experiment 2, a total of 566 White Mirthys pigeon squabs were randomly assigned to three treatment groups: W28, W7, and W0. These groups represent squabs separated from their parents at different ages: 28 days, 7 days, and 0 days (at hatch), respectively.

**Results:**

The results indicated that early weaning of squabs significantly decreased body weight, weight gain, feed intake, and feed conversion ratio (*P* < 0.05), while also increasing the mortality rate in the W0 group compared to the W28 and W7 groups. There were no significant differences in body weight, weight gain, or mortality rate between the W28 and W7 groups. Pigeons in the W0 group exhibited significantly lower (*P* < 0.001) egg-laying cycle and reproductive cycle. Still, they produced more eggs laid and weaned squabs than the W28 and W7 groups. Early weaning had no significant impact on hatchability rate (*P* = 0.220), egg weight (*P* = 0.580), egg length (*P* = 0.308), egg width (*P* = 0.488), or egg shape index (*P* = 0.167). However, the eggs from the early weaning group (W0) had a lower shell thickness (*P* = 0.002) compared to the control (W28) and W7 groups.

**Conclusions:**

Early weaning at hatching has been found to reduce the growth performance of squabs; however, it significantly enhances the reproductive performance of parent pigeons. This method presents a promising strategy for increasing the reproductive rate of parent pigeons and boosting the annual production of squabs.

## Background

Pigeons (*Columba livia*) are recognized as one of the first bird species domesticated by humans, dating back over 3,500 years [1]. Initially, they were used for religious rituals, conveying messages, and later became a food source [2, 3]. Pigeon meat is considered a high-quality food option; it is low in cholesterol, rich in protein, and possesses a distinctive flavor and aroma [4]. In Egypt, pigeon meat is regarded as a delicacy of high value [5]. Pigeon farming for meat has also gained in the United States [6], as well as in Europe and Asia [7, 8]. Additionally, pigeon eggs are a staple food in many cultures. They contain high-quality protein and many essential nutrients [9–11].

Traditional pigeon breeding has evolved into commercial or industrial farms in response to the growing demand for their meat. However, the pigeon meat production sector is distinct from other poultry industries because of its different growth characteristics [12, 13]. Squabs are superaltricial, completely depend on their parents for feeding and care during this early stage of life, until they are weaned [14]. In the traditional breeding method, squabs are usually weaned while remaining in their nests, a process that generally takes about 21 to 28 days after hatching [15–17]. During this period, the parent pigeons care for squabs [14], and they are unable to enter a new egg-laying cycle.

The egg-laying cycle significantly influences the production of pigeons. A breeding pair of pigeons produces about 12 squabs annually through natural weaning, each squab weighs between 450 and 700 g [18]. This production rate is considered low compared to other poultry species. The egg-laying cycle for local Egyptian pigeons is approximately 52.75 days [19]; while for local Indonesian pigeons, it is around 51 days [20]. The silver king pigeon breed has an egg-laying cycle of 39.2 days [17], and local Bangladeshi pigeons have a cycle of 37.17 days [21]. Ahmed [22] reported that the period between hatching and next laying in the White Mirthys breed is 19.50 days.

The traditional breeding system for pigeons is not well-suited for commercial or industrial production, as it limits squab output and hinders the ability to maximize economic returns. To improve production efficiency, it is essential to artificially feed squabs that have been weaned early [23]. Previous studies indicate that early weaning can be used. For instance, Wen et al. [23] reported that domestic pigeon squabs can be weaned as early as the seventh day. Similarly, Abdel-Azim et al. [24] found that pigeon chicks were weaned as early as the third day after hatching and were then hand-fed with crop milk substitutes. For the effect of early weaning of squabs on maternal productivity, if young pigeons are weaned early, the dam can lay eggs 10 to 20 days earlier than usual [24]. This could increase the egg production for each pair of pigeons from 6 pairs to between 10 and 15 pairs per year [25]. While early weaning may negatively affect the body weight and growth rate of squabs [23], it presents a promising strategy to enhance the reproductive rate of parent stock by increasing the annual production of squabs [24]. Currently, squabs are weaned on a large scale in commercial farms immediately after hatching. They are fed a crop milk replacer by hand, which allows their parents to continue laying eggs, thereby increasing the number of eggs and squabs produced. Additionally, to our knowledge, no detailed studies have been previously reported on the effects of weaning at hatching on either the squabs or their parents. Therefore, this study was conducted to evaluate the effects of early weaning of squabs at 0 and 7 days old on growth performance, feed consumption, reproductive performance of the parents, and egg traits.

## Materials and methods

The current study was conducted in a commercial pigeon farm, located on the Cairo-Alexandria Desert Road, Alexandria, Egypt.

### Experimental design

Two experiments were conducted to evaluate the effects of early weaning on growth performance, feed consumption, reproductive performance of the parents, and egg traits for the White Mirthys pigeon.

In experiment 1, a total of 300 pairs of adult White Mirthys pigeons were randomly divided into three groups: W28, W7, and W0. These groups correspond to the ages at which the pigeons were separated from their squabs: 28 days, 7 days, and 0 days (at hatch), respectively. Each group consisted of ten replicates, with each replicate comprising ten pairs of pigeons.

In experiment 2, a total of 566 White Mirthys pigeon squabs were randomly assigned to the same three treatment groups: W28, W7, and W0, representing separation from their parents at 28 days, 7 days, and 0 days (at hatch), respectively.

### Bird housing

Each pair of parent pigeons, consisting of sire and dame, was housed as an individual family in numbered metal cages. Each cage measured 60 cm in width, 60 cm in length, and 50 cm in height, and included two nests. The cages were arranged in an open-house system that maintained hygienic conditions with natural ventilation. To provide adequate light, artificial lighting was used to ensure a total of 17 h of light per day. For artificial incubation in the W0 group, newly hatched squabs are placed in a controlled brooding area set at 35 °C and 60% humidity on the first day after hatching. The temperature is gradually reduced by 1 °C each subsequent day until it reaches 30 °C, which is maintained until the feathers cover the squabs sufficiently. Thereafter, the temperature is further decreased based on feather development until it stabilized at room temperature (approximately 22–24 °C) by the time squabs reached 14–16 days of age.

### Experimental diets

The parental pigeons received pellet feed twice daily (17.3% protein and energy content of 12.8 MJ/ kg) and had free access to feed and water. The squabs in the W28 group were fed by the parents from hatching until 28 days old. The squabs in the W7 group were fed by their parents for the first 7 days after hatching and then were hand-fed with mashed feed from days 8 to 28. The squabs in the W0 group were hand-fed with crop milk replacer (40.6% protein and energy content of 14.5 MJ/ kg) for the first 7 days after hatching four times daily and then were hand-fed with mashed feed from days 8 to 28. Crop milk replacer is a commercial powdered product that is mixed with warm water (37 °C) in a ratio of 1:2 and administered using a syringe prepared specifically for feeding young birds. The mash feed is the same as the pelleted feed given to adult pigeons and is prepared by grinding, mixing with warm water (37 °C), and hand feeding to the squabs from 8 to 28 days of age.

### Growth performance

Squabs were weighed at days 0 (initial body weight), 7, and 28 (final body weight). Based on these measurements, weight gain during different periods and the feed conversion ratio were calculated with the following formulas:

Weight gain (g) = final body weight − initial body weight.

Feed conversion ratio (FCR) = total feed intake (g) ÷ total weight gain (g).

The mortality rate is the percentage of deaths in squabs, calculated for all treatment groups from hatching until 28 days. The total amount of feed consumed by each pair was calculated in all treatments from hatching to 28 days.

### Reproductive performance

The egg laying cycle was calculated as the interval between fertilized egg deposits in consecutive clutches. The reproductive cycle was calculated as the days between two successive weaning. The number of eggs and weaned squabs were calculated for each pair over a period of 160 days. The length and width of each egg were measured, and the egg shape index (%) was calculated by dividing the width by the length and multiplying by 100. Eggs from the W0 group were artificially incubated using a PTO egg hatching machine (Model C2). During the incubation phase, the temperature was consistently maintained at 37.3 °C, and the humidity was kept at 60% according to Vatnick [26]. For the first 15 days, the eggs were turned every two hours. After this period, they were moved to the hatching tray and placed in individual breeding boxes, which are used to ensure that paternity is accurately determined for each squab. The thickness of the eggshell was measured after hatching using a micrometer. Total hatchability percentage is calculated by dividing hatched eggs by incubated eggs.

### Statistical analysis

A preliminary analysis was conducted using Jamovi 2.2.5 software (The Jamovi Project, 2022) to obtain least-squares means. The effects of early weaning ages, which included three treatments, were treated as fixed effects according to the model Yij = µ + Tj + eij, where Yij represents the dependent variable for each observation, µ is the overall mean, Tj denotes the effect of treatment j, and eij represents the error term associated with each observation. An analysis of variance (ANOVA) was performed, followed by Tukey’s test to determine significant differences among the treatments. The level for statistical significance was set at *p* < 0.05.

## Results

### Growth performance

The effects of early weaning of squabs at 0 and 7 days of age on body weight, weight gain, mortality rate, feed intake, and feed conversion ratio are presented in Table 1. There was no significant differences (*P* = 0.502) in initial body weight between the groups. However, at both 7 days of age and final body weight, the body weight was lower (*P* < 0.001) in the W0 group compared to the W28 and W7 groups. Weight gain in the W0 group was also significantly lower from 0 to 7 days (*P* < 0.001), from 7 to 28 days (*P* < 0.001), and from 0 to 28 days (*P* = 0.005) when compared to the W28 and W7 groups. The mortality rate for squabs was higher in the W0 group (*P* < 0.001) compared to the W28 and W7 groups. The total feed intake (g/ pair of squabs) was the lowest in the W0 group (*P* = 0.034), followed by the W7 group, and then the W28 group. The W0 group had the best feed conversion ratio (*P* = 0.016), followed by the W7 group and then the W28 group.

**Table 1:** Effect of early weaning on growth performance, feed consumption, mortality rate, and feed conversion ratio

Items	Groups			SEM	*P*-value
	W28	W7	W0		
Initial body weight, g/ squab	17.43	17.61	17.55	0.27	0.502
Body weight at d 7, g/ squab	157 ^a^	161 ^a^	125 ^b^	3.01	< 0 0.001
Final Body weight, g/ squab	458 ^a^	465 ^a^	367 ^b^	8.15	< 0 0.001
Weight gain at 0–7 d of age, g / squab	132 ^a^	134 ^a^	102 ^b^	4.03	< 0 0.001
Weight gain at 7–28 d of age, g / squab	309 ^a^	293 ^a^	241 ^b^	7.33	< 0 0.001
Weight gain at 0–28 d of age, g / squab	442 ^a^	446 ^a^	349 ^b^	21.24	0.005
Mortality rate, %	3.80 ^b^	3.28 ^b^	26.70 ^a^	0.51	< 0 0.001
Total feed intake, g/pair of squabs	3220 ^a^	2805 ^b^	2033 ^c^	41.0	0.034
Feed conversion ratio (g feed/ g gain)	3.66 ^a^	3.19 ^b^	2.95 ^c^	0.21	0.016

W28: weaning group after 28 d of hatching; W7: weaning group after 7 d of hatching; W0: weaning group after 0 d of hatching

^a, b, c^ Means within the same row with different superscripts differ significantly (*P* < 0.05)

SEM: standard error of the mean

### Reproductive performance and egg traits

The effects of early weaning of squabs at 0 and 7 days of age on reproductive performance and egg traits are shown in Table 2. Pigeons in the W0 group showed a significantly shorter egg laying and reproductive cycle and significantly higher number of eggs in 160 days/pair (*P* < 0.001) compared to the W28 and W7 groups. There was no significant effects of early weaning on hatchability rate (*P* = 0.220), egg weight (*P* = 0.580), egg length (*P* = 0.308), egg width (*P* = 0.488), or egg shape index (*P* = 0.167). However, eggs from the early weaning group (EW0) had a lower shell thickness (*P* = 0.002) compared to the control (W28) and W7 groups.

**Table 2:** Effect of early weaning on reproductive performance and egg traits

Items	Groups			SEM	*P*-value
	W28	W7	W0		
Egg laying cycle, days	38.1 ^a^	37.5 ^a^	10.2 ^b^	0.54	< 0 0.001
Reproductive cycle, days	38.1^a^	38.3 ^a^	11.6 ^b^	0.78	< 0 0.001
laying rate in 160 days/ pair	8 ^b^	8.59 ^b^	16.71 ^a^	0.51	< 0 0.001
Hatchability rate, %	94.93	95.01	94.86	0.13	0.220
Number of weaned squabs over 160 days/ pair	7.38 ^b^	7.87 ^b^	12.44 ^a^	0. 25	< 0.001
Egg weight, g	19.93	19.87	20.00	0.18	0.580
Egg length, cm	3.92	3.94	3.91	0.02	0.308
Egg width, cm	2.85	2.94	2.94	0.01	0.488
Egg shape index	73.18	74.30	74.09	0.15	0.167
Shell thickness, mm	0.198^a^	0.195 ^a^	0.171^b^	0.01	0.002

W28: weaning group after 28 d of hatching; W7: weaning group after 7 d of hatching; W0: weaning group after 0 d of hatching

^a, b^ Means within the same row with different superscripts differ significantly (*P* < 0.05)

SEM: standard error of the mean.

## Discussion

### Growth performance

Pigeons are birds that cannot feed themselves for a certain period after hatching. During this period, they depend entirely on a special type of feed produced by the crop of the parent pigeons, known as crop milk [16, 27, 28]. This crop milk nourishes the squabs until they can eat independently. The crop milk primarily consists of fats and proteins [29, 30]. Additionally, it contains a significant amount of maternal antibodies [13, 31]. Consequently, the growth and development of squabs rely heavily on crop milk [23, 32]. Our results indicate that early weaning from hatch negatively affects growth performance. This finding aligns with observations obtained by Wen et al. [23]. The reduction in body weight and weight gain observed in the W0 group of squabs may be attributed to the differing nutritional values between natural crop milk and commercial crop milk replacers. On the other hand, our study suggests that a slight delay in weaning of squabs to the 7-day-old could help mitigate some of the negative effects associated with early weaning. Previous studies have shown that early weaning adversely affects the length and weight indices of the small intestine, including the duodenum, jejunum, and ileum. This is particularly evident in the ileum, where early weaning leads to a decrease in length [33–35]. This could explain the significant reduction in body weight in birds weaned at hatch. Other studies indicated that providing antioxidants and nutritional supplements can help rabbits and piglets endure a less stressful weaning process by promoting the lengthening of their small intestine [36–38]. If similar approaches are applied to pigeons, it may help minimize the negative effects of early weaning on growth performance in squabs and enhance the reproductive traits of parent birds.

The findings of this study indicate that early weaning at hatch (W0) significantly increases mortality rates among young pigeons. In contrast, early weaning after 7 days (W7) post-hatching shows similar outcomes to natural weaning (W28). While early weaning at hatch is considered a modern approach to enhancing pigeon production, it necessitates further research to ensure proper care and nutrition, thereby reducing mortality rates. The high mortality rates observed in the squabs from the W0 group may be attributed to early weaning. This practice disrupts gut development, influencing the length and weight indices of the ileum [25]. It also adversely affects small intestine health, including enzyme activity, antioxidant status, and inflammatory cytokine levels [25], all of which are crucial for digestion and nutrient absorption. Consequently, these disruptions can lead to increased mortality in the squabs. These findings align with previous studies by Smith et al., [39], Meale et al., [40], Chen et al., [41], Li et al., [42], and Xu et al., [43] which also highlighted the negative effects of early weaning in animals, often resulting in heightened mortality rates.

The results of this study indicate that the birds in the W0 group had a feed intake that was 38.9% lower than that of the W28 group and 27.5% lower than the W7 group. Furthermore, the W0 group exhibited the greatest improvement in feed conversion rate, followed by the W7 group, with the W28 group showing the least improvement.

### Reproductive performance and egg traits

To enhance pigeon productivity for commercial purposes, one strategic goal is to reduce the egg-laying and reproductive cycles of parent pigeons. This approach aims to increase the number of eggs available for artificial incubation. Our results indicated that early weaning at hatch (W0) positively affects the egg-laying and reproductive cycles compared to early weaning at 7 days of age (W7) or a control group. The significant decrease in the reproductive cycle due to early weaning at hatch could lead to increased productivity by increasing the number of squabs produced each year per pair of parent pigeons. During the incubation period, parent pigeons care for their eggs and squabs, which prevents them from entering a new egg-laying cycle. It has been suggested that prolactin levels associated with this incubation behaviour may explain the differences in egg laying cycle and reproductive cycle among various groups. Mohamed et al. [44] reported fluctuations in prolactin levels throughout the reproductive cycle, with levels peaking during egg incubation and declining after hatching. Since high prolactin levels are linked to the timing of egg laying, these fluctuations may help explain variations in mating frequency and egg production [45, 46].

Early weaning at hatching increased the number of eggs produced per pair during the experimental period compared to both the 7-day weaning group and the control group. However, early weaning did not affect egg weight, length, width, or shape index. Early weaning resulted in a higher number of mating between dames and sires. This finding aligns with those of Al-Sagheer et al. [47], who reported that mating rates did not influence egg production traits in broiler chickens. Conversely, early weaning at hatching significantly reduced eggshell thickness compared to the other groups. This decrease may be attributed to the higher egg production observed in the W0 group of parent pigeons. This finding aligns with Roberts’ [48] observation that several factors, including bird age, breed, production system, and general stress, affect eggshell quality, particularly its strength and thickness.

## Conclusion

It can be concluded that Early weaning of squabs at hatching decreased their body weight, growth, and feed intake while increasing squab mortality. The pigeons in the W0 group experienced a shorter egg-laying and breeding cycle, produced more eggs, and successfully weaned more squabs. Early weaning did not have a significant effect on hatching rate, egg weight, egg length, egg width, or egg shape index. However, eggs from the early weaning group did have a lower shell thickness. Overall, while early weaning at hatching was found to reduce the growth performance of the squabs, it significantly improved the reproductive performance of the parent pigeons. This approach presents a promising strategy for increasing the reproductive rate of parent pigeons and enhancing annual egg production. Further studies are needed to decrease squab mortality associated with artificial incubation. This can be achieved by identifying optimal diets enhanced with feed additives and placing the eggs under a less valuable pigeon to take over the incubation and feeding process. Additionally, it is important to evaluate the effect of artificial incubation on the quality of the squabs.

## Data Availability

Datasets generated and/or analyzed during this study are included in this article version, and if required any further information related to the data involved in the manuscript can be obtained from the corresponding author upon reasonable request.

## References

[1] Stringham SA, Mulroy EE, Xing J, Record D, Guernsey MW, Aldenhoven JT, Osborne EJ, Shapiro MD. Divergence, convergence, and the ancestry of feral populations in the domestic rock pigeon. Curr Biol. 2012;22:302–8. 22264611 10.1016/j.cub.2011.12.045 PMC3288640

[2] Kokoszyński D, Stęczny K, Żochowska-Kujawska J, Sobczak M, Kotowicz M, Saleh M, Fik M, Arpášová H, Hrnčár C, Włodarczyk K. Carcass characteristics, physicochemical properties, and texture and microstructure of the meat and internal organs of carrier and King pigeons. Animals. 2020;10:1315. 32751657 10.3390/ani10081315 PMC7459732

[3] Chang L, Tang O, Zhang R, Fu S, Mu C, Shen X, Bu Z. Evaluation of meat quality of local pigeon varieties in China. Animals. 2023;13:1291. 37106854 10.3390/ani13081291 PMC10135284

[4] Pomianowski JF, Mikulski D, Pudyszak K, Cooper RG, Angowski M, Jóźwik A, Horbańczuk JO. Chemical composition, cholesterol content, and fatty acid profile of pigeon meat as influenced by meat-type breeds. Poult Sci. 2009;88(6):1306–9. 19439644 10.3382/ps.2008-00217

[5] Omar AS, Abd ESA, Abdel-Aziz YAA, Sammour HB, Aggour MG. A field study on pigeon production systems in the rural sector of El-Sharkia Governorate, Egypt. Egypt Poult Sci J. 2014;4:1039–53.

[6] Kabir MA. Limitations in pigeon keeping: A review. J Multidiscip Appl Nat Sci. 2021;2:100–5.

[7] Liu R, Xing L, Zhou G, Zhang W. What is meat in China? Anim. Front. 2017;4:53–6.

[8] Akter MTD, Sarder MJU, Islam MH, Islam MR, Parvez NH, Alam MN. Evaluation of the productive and reproductive performance of pigeon in selected districts of Bangladesh. Clin Podiatr Med Sur. 2020;1:1–4.

[9] Damaziak K, Kieliszek M, Bucław M. Characterization of structure and protein of vitelline membranes of precocial (ring-necked pheasant, Gray partridge) and superaltricial (cockatiel Parrot, domestic pigeon) birds. PLoS ONE. 2020;15(1):e0228310. 31999757 10.1371/journal.pone.0228310 PMC6992205

[10] Xiao Y, Zhao Z, Zhang T, Xu X, Anik K, Qiu Y, Xu Z, Li S, Xu H. A new glycoprotein from pigeon egg: study on its structure and digestive characteristics. Food Res Int. 2024;194:114875. 39232513 10.1016/j.foodres.2024.114875

[11] Hu G, Yang C, He H, Li S, Xiang X, Harlina PW, Wang J, Geng F. Comprehensive analysis of pigeon egg proteins: composition, function, and health significance. J Food Comp Anal. 2024;126:105941.

[12] Gao CQ, Wang XH, Hu XC, Yan HC, Wang XQ. Effects of dietary crude protein levels on growth performance, carcass characteristics, meat quality of squabs and laying performance of breeding pigeons (in Chinese). J South China Agric Univ. 2016;37(4):1–6.

[13] Jin C, He Y, Jiang S, Wang X, Yan H, Tan H, Gao C. Chemical composition of pigeon crop milk and factors affecting its production: a review. Poult Sci. 2023;102(6):102681. 37098298 10.1016/j.psj.2023.102681 PMC10149254

[14] Horseman ND, Buntin JD. Regulation of pigeon crop-milk secretion and parental behaviors by prolactin. Annu Rev Nutr. 1995;15(1):213–38. 8527218 10.1146/annurev.nu.15.070195.001241

[15] Sales J, Janssens GPJ. Nutrition of the domestic pigeon (*Columba Livia domestica*). J World’s Poult Sci. 2003;59(2):221–32. 10.1093/ps/82.9.1457 12967260

[16] Mahdy MA. Comparative morphological study of the oropharyngeal floor of squabs and adult domestic pigeons (*Columba Livia domestica*). Microsc Res Tech. 2021;84(3):499–511. 32959459 10.1002/jemt.23606

[17] Ji F, Zhang S, An Y, Wang Z, Shao Y, Du S, Xing L, Sun X. Influence of dietary phosphorus concentrations on the performance of rearing pigeons (*Columba livia*), and bone properties of squabs. Poult Sci. 2022;101(4):101744. 35220034 10.1016/j.psj.2022.101744 PMC8881650

[18] Jilly B. Development opportunities and profitability of private utility pigeon breeding in Hungary. Hung Agric Res. 2018;27:20–5.

[19] Abou Khashaba HA, Mariey YA, Sayed HAM. Nutritional and management studies on the pigeon: estimate of protein requirements. J Anim Poult Prod. 2008;33(12):8447–61.

[20] Darwati S, Martojo H, Cece Sumantri, Sihombing DTH, Mardiastuti A. Productivity, repeatability of productive and reproductive traits of local pigeon. J Indones Trop Anim Agric. 2010;35(4):268–74.

[21] Kabir MA. Productivity of crossed Indigenous pigeon in semi intensive system. Res J Agric Sci. 2013;2(1):1–4.

[22] Ahmed O. Evaluation of some productive and reproductive traits of three pigeon strains. PhD thesis, Matrouh University. 2023.

[23] Wen JS, Xu QQ, Zhao WY, Hu CH, Zou XT, Dong XY. Effects of early weaning on intestinal morphology, digestive enzyme activity, antioxidant status, and cytokine status in domestic pigeon squabs (*Columba livia*). Poult Sci. 2022;101(2):101613. 34936957 10.1016/j.psj.2021.101613 PMC8703073

[24] Abdel-Azeem AF, Amer AA, Shama TA, Abbas WA. Early weaning of pigeon squabs. Egypt Poult Sci J. 2016;36(1):205–32.

[25] Xu Q, Jian H, Zhao W, Li J, Zou X, Dong X. Early weaning stress induces intestinal microbiota disturbance, mucosal barrier dysfunction and inflammation response activation in pigeon squabs. Front Microbiol. 2022;13:877866. 35711747 10.3389/fmicb.2022.877866 PMC9194612

[26] Vatnick I, Foertsch S. Incubation temperature of the pigeon embryo (*Columba livia*). J Therm Biol. 1998;23(1):53–7.

[27] Ma H, Ni A, Ge P, Li Y, Shi L, Wang P, Fan J, Isa AM, Sun Y. hen J. Analysis of long non-Coding RNAs and mRNAs associated with lactation in the crop of pigeons (*Columba livia*). Genes. 2020;11(2):201. 10.3390/genes11020201 PMC7073620 32079139

[28] Shao Y, Ma W, Ji F, Sun X, Du S, Li X, Li Q, Wang Z. Exploration of proteomics analysis of crop milk in pigeons (*Columba livia*) during the lactation period. ACS Omega. 2021;6(42):27726–36. 34722973 10.1021/acsomega.1c02977 PMC8552352

[29] Hegde SN. Composition of pigeon milk and its effect on growth in chicks. Indian J Exp Biol. 1973;11(3):238–9. 4782628

[30] Ding J, Liao N, Zheng Y, Yang L, Zhou H, Xu K, Han C, Luo H, Qin C, Tang C, Wei L. The composition and function of pigeon milk microbiota transmitted from parent pigeons to squabs. Front Microbiol. 2020;11:1789. 32849405 10.3389/fmicb.2020.01789 PMC7417789

[31] Bharathi L, Shenoy KB, Hegde SN. Biochemical differences between crop tissue and crop milk of pigeons (*Columba livia*). Comp Biochem Physiol A. 1997;116(1):51–5.

[32] Zhao W, Liu Q, Jiang H, Zheng M, Qian M, Zeng X, Bai W. Monitoring the variations in physicochemical characteristics of squab meat during the braising cooking process. Food Sci Nutr. 2022;10(8):2727–35. 35959272 10.1002/fsn3.2876 PMC9361449

[33] King MR, Morel PCH, Revell DK, Pluske JR, Birtles MJ. Dietary bovine colostrum increases villus height and decreases small intestine weight in early-weaned pigs. Asian-Australas J Anim Sci. 2008;21(4):567–73.

[34] Wang M, Yang C, Wang QY, Li JZ, Li YL, Ding XQ, Yin J, Yang HS, Yin YL. The growth performance, intestinal digestive and absorptive capabilities in piglets with different lengths of small intestines. Animal. 2020;14(6):1196–203. 31829913 10.1017/S175173111900288X

[35] Liu G, Zheng J, Wu X, Xu X, Jia G, Zhao H, Chen X, Wu C, Tian G, Wang J. Putrescine enhances intestinal immune function and regulates intestinal bacteria in weaning piglets. Food Funct. 2019;10(7):4134–42. 31241125 10.1039/c9fo00842j

[36] Zheng P, Yu B, He J, Tian G, Luo Y, Mao X, Zhang K, Che L, Chen D. Protective effects of dietary arginine supplementation against oxidative stress in weaned piglets. Br J Nutr. 2013;109(12):2253–60. 23176743 10.1017/S0007114512004321

[37] Xu J, Xu C, Chen X, Cai X, Yang S, Sheng Y, Wang T. Regulation of an antioxidant blend on intestinal redox status and major microbiota in early weaned piglets. Nutr. 2014;30(5):584–9. 10.1016/j.nut.2013.10.018 24698350

[38] Liu L, Zuo W, Li F. Dietary addition of Artemisia argyi reduces diarrhea and modulates the gut immune function without affecting growth performances of rabbits after weaning. J Anim Sci. 2019b;97(4):1693–700. 30726960 10.1093/jas/skz047 PMC6447264

[39] Smith F, Clark JE, Overman BL, Tozel CC, Huang JH, Rivier JE, Blisklager AT, Moeser AJ. Early weaning stress impairs development of mucosal barrier function in the Porcine intestine. Am J Physiol Gastrointest Liver Physiol. 2010;298(3):G352–63. 19926814 10.1152/ajpgi.00081.2009 PMC2838512

[40] Meale SJ, Li S, Azevedo P, Derakhshani H, Plaizier JC, Khafipour E, Steele MA. Development of ruminal and fecal microbiomes are affected by weaning but not weaning strategy in dairy calves. Front Microbiol. 2016;7:582. 27199916 10.3389/fmicb.2016.00582 PMC4853645

[41] Chen L, Xu Y, Chen X, Fang C, Zhao L, Chen F. The maturing development of gut microbiota in commercial piglets during the weaning transition. Front Microbiol. 2017;8:1688. 28928724 10.3389/fmicb.2017.01688 PMC5591375

[42] Li C, Wang W, Liu T, Zhang Q, Wang G, Li F, Li F, Yue X, Li T. Effect of early weaning on the intestinal microbiota and expression of genes related to barrier function in lambs. Front Microbiol. 2018;9:1431. 30013534 10.3389/fmicb.2018.01431 PMC6036172

[43] Xu QQ, Zhang XY, Zou XT, Dong XY. Effects of in Ovo injection of L-histidine on hatch performance and post-hatch development in domestic pigeons (*Columba livia*). Poult Sci. 2019;98:3194–203. 30753623 10.3382/ps/pez046

[44] Mohamed RA, Shukry M, Mousa-Balabel TM, Elbassiouny AA. Assessment of plasma prolactin and nest defense behaviour during breeding cycle of pigeon (*Columba Livia domestica*). J Environ Agric Sci. 2016;7(19):19–22.

[45] Bouaouiche A, Salah BM, Abdennour C. Influence of photoperiod and prolactin on reproductive pigeons *Columba Livia domestica*. Eur J Sci Res. 2009;36(2):280–4.

[46] Dong XY, Wang YM, Song HH, Zou XT. Effects of in Ovo injection of carbohydrate solution on small intestine development in domestic pigeons (*Columba livia*). J Anim Sci. 2013;91(8):3742–9. 23658350 10.2527/jas.2013-6400

[47] Elsagheer MA, Mohamed OA, Khattab IM, Wadea MK. Impact of different mating ratios of broiler breeder on reproductive performance during post moult phase. Anim Biotechnol. 2024;35(1):2398707. 39222029 10.1080/10495398.2024.2398707 PMC12674434

[48] Roberts JR. Factors affecting egg internal quality and egg shell quality in laying hens. Poult Sci J. 2004;41(3):161–77.

